# Control of Citrus Post-harvest Green Molds, Blue Molds, and Sour Rot by the Cecropin A-Melittin Hybrid Peptide BP21

**DOI:** 10.3389/fmicb.2018.02455

**Published:** 2018-10-10

**Authors:** Wenjun Wang, Sha Liu, Lili Deng, Jian Ming, Shixiang Yao, Kaifang Zeng

**Affiliations:** ^1^College of Food Science, Southwest University, Chongqing, China; ^2^Research Center of Food Storage & Logistics, Southwest University, Chongqing, China

**Keywords:** peptide BP21, post-harvest, citrus fruit, diseases control, mode of action

## Abstract

In this study, the activity of the cecropin A-melittin hybrid peptide BP21 (Ac-FKLFKKILKVL-NH_2_) in controlling of citrus post-harvest green and blue molds and sour rot and its involved mechanism was studied. The minimum inhibitory concentrations of BP21 against *Penicillium digitatum*, *Penicillium italicum*, and *Geotrichum candidum* were 8, 8, and 4 μmol L^-1^, respectively. BP21 could inhibit the growth of mycelia, the scanning electron microscopy results clearly showed that the mycelia treated with BP21 shrank, formed a rough surface, became distorted and collapsed. Fluorescent staining with SYTOX Green (SG) indicated that BP21 could disintegrate membranes. Membrane permeability parameters, including extracellular conductivity, the leakage of potassium ions, and the release of cellular constituents, visibly increased as the BP21 concentration increased. Gross and irreversible damage to the cytoplasm and membranes was observed. There was a positive correlation between hemolytic activity and the concentration of BP21. These results suggest peptide BP21 could be used to control citrus post-harvest diseases.

## Introduction

Green mold, blue mold, and sour rot caused by *Penicillium digitatum*, *Penicillium Italicum*, and *Geotrichum candidum* (syn. *Geotrichum citri-aurantii*) are the most serious post-harvest fungal diseases. Sour rot cannot be inhibited by imazalil and thiabendazole, which are effective chemical fungicides against green mold and blue mold (Droby et al., 2002; Ismail and Zhang, 2004). The application of chemical fungicides has been restricted due to the concerns about pesticide residues, environmental pollution and pathogens resistance. It is urgent to search for effective, ecofriendly methods of diseases control to replace or reduce the use of harmful chemical fungicides (Schirra et al., 2011; Romanazzi et al., 2017).

Antimicrobial peptides (AMPs) as novel antibiotics are widely studied, it has been proposed their use to fight phytopathogens in agriculture, animal husbandry, post-harvest conservation, and the food industry (Jenssen et al., 2006; Keymanesh et al., 2009; Ciociola et al., 2016). The application of peptides in the control of fruit and vegetable diseases is gaining attention. An increasing number of AMPs have been shown to control fruit and vegetable diseases. In previous research, PAF56 (GHRKKWFW) was shown to effectively control of fungi infection in citrusfruits (Wang et al., 2018). There are likely many AMPs yet to be discovered that can effectively control fruit and vegetable diseases.

Cecropins were first discovered in the hemolymph of the giant silk moth *Hyalophora cecropia* (Andreu et al., 1983), and they are some of the best known cationic AMPs, representing a family of highly basic α-helical peptides. In particular, Cecropin A displays powerful lytic activity against bacteria but has no cytotoxic effects against eukaryotic cells. However, because many fruit and vegetable diseases are caused by fungi, certain natural AMPs should be modified with new sequences that confer improved antimicrobial and therapeutic properties (Chicharro et al., 2001; Alberola et al., 2004). In particular, certain peptides from the CECMEL11 library (LIPPSO-CIDSAV, University of Girona, Girona, Spain), which is composed of *de novo* designed and synthetically produced cecropin A-melittin hybrid linear undecapeptides, have been derived from the peptide Pep3 (WKLFKKILKVL-NH_2_) and evaluated for *Stemphylium vesicarium* infection control in pears (Badosa et al., 2009). For example, BP15 (KKLFKKILKVL-NH_2_) inhibited *S. vesicarium* growth, produced morphological alterations to germ tubes and induced cell membrane disruption (Ferre et al., 2006; Puig et al., 2014, 2016). BP15 also could control the infection caused by *P. digitatum* on citrus fruit (Muñoz et al., 2007). In addition, BP21 (Ac-FKLFKKILKVL-NH_2_) was designed to inhibit the plant pathogenic fungi *Fusarium oxysporum*, *Aspergillus niger*, *Rhizopus stolonifer*, and *Penicillium expansum.* It has been shown that BP21 can effectively inhibit *P. expansum in vitro* and control the post-harvest decay caused by *P. expansum* in apples (Badosa et al., 2009). *Penicillium* species that affect the post-harvest of fruits further highlight the need to develop AMPs. We predicted that BP21 could control post-harvest diseases on citrus fruit as well. Cecropin A, melittin and their hybrids have been widely studied for their antibacterial mode of action (Makovitzki et al., 2007; Ferre et al., 2009), but the mechanisms that underlie their interactions with plant pathogenic filamentous fungi are still unclear.

The aim of the present study was to investigate the effects of the peptide BP21 in inhibiting *P. digitatum, P. italicum*, and *G. candidum in vitro* and *in vivo*, and the mode of action of BP21 was studied.

## Materials and Methods

### Antifungal Peptide and Fungal Strains

Peptide BP21 (FKLFKKILKVL) was synthesized at >90% purity from GenScript Corporation (Nanjing, China) by solid-phase methods using *N*-(9-fluorenyl) methoxycarbonyl (Fmoc) chemistry. BP21 was acetylated at the N terminus (Ac) and amidated at the C terminus (NH_2_). Stock solutions of peptides were prepared at 1 m mol L^-1^ in sterile ultrapure water and stored at -40°C.

The fungi (*P. digitatum*, *P. italicum*, and *G. candidum*) used in this work were obtained from spoiled citrus fruits and identified. They were cultured on potato dextrose agar (PDA) that contained an infusion of 200 g L^-1^ potatoes, 20 g L^-1^ glucose, and 20 g L^-1^ agar at 25°C. The spores from a 7-day-old culture were collected, filtered, and adjusted to the suitable concentration with the aid of a hematocytometer (Jeong et al., 2016).

### Effects of the Fungal Growth *in vitro*

The fungicidal activities of the peptide BP21 was determined by dose–response curves as previously described (López-García et al., 2002; Wang et al., 2018). BP21 was added to a final concentration of 0.25, 0.5, 1, 2, 4, 8, 16, 32, 64 μM, respectively. In all experiments, three replicates were prepared for each treatment. The growth of the fungi was determined by measuring OD_600_ using a Multiskan Spectrum microplate spectrophotometer (BioTek Instruments, Inc., United States) at 48 h after mixing with BP21. The minimum inhibitory concentration (MIC) of the peptide BP21 for three fungi was defined as the peptide BP21 concentration that completely inhibited growth in all the experiments carried out.

### Scanning Electron Microscopy (SEM)

To determine the effect of BP21 on the mycelia morphology of the three fungi, a scanning electron microscopic (SEM) study was performed. The mycelia from 2-day-old culture were collected, washed, and then resuspended in sterilized distilled water. BP21 (10 or 100 μmol L^-1^) was mixed into the suspensions for 48 h, and controls without BP21 were tested similarly. The mycelia were collected and placed in vials containing 3.0% (v/v) glutaraldehyde in 0.05 mol L^-1^ phosphate buffered saline (pH 6.8) at 4°C. The mycelia were kept in this solution for 48 h for fixation and then washed with 0.05 mol L^-1^ phosphate buffered saline two times. The samples were dehydrated in an ethanol series (30, 50, 70, 85 and 95%, v/v) for 10 min in each alcohol dilution, ending with absolute ethanol twice. Then, the ethanol was replaced with tertiary butyl alcohol. After dehydration, the samples were dried with carbon dioxide. Finally, the specimens were sputter-coated with gold in an ion coater for 2 min. All samples were viewed in a JEOL JSM-6510LV SEM (JEOL, Tokyo, Japan) operating at 25 kV at 5000× magnification (Tao et al., 2014).

### Fluorescence Microscopy

The mode of action of BP21 with the mycelia was characterized by the fluorescent dye SYTOX Green (SG) (Molecular Probes; Invitrogen, Corp, Carlsbad, CA, United States) as described previously (Puig et al., 2016; Wang et al., 2018). BP21 was used to each treatment groups to reach a final concentration of 10 or 100 μmol L^-1^. After incubation with BP21, the fungal suspensions were stained with SG. Fluorescence was examined and photographed with an Eclipse TS100 epifluorescence microscope (Nikon Corporation, Japan) with FITC filter sets. Simultaneous brightfield images were captured as well.

### Measurement of Extracellular Conductivity, K^+^ Efflux and Release of Cellular Constituents

BP21 was used to each treatment groups to reach a final concentration of 10 or 100 μmol L^-1^. The extracellular conductivity of mycelia by using a DDS-307A conductivity meter (INESA, Shanghai, China) according to a previously described method (Wang et al., 2018), and controls without BP21 were tested similarly. Then, a previously described method was used to determine the amount of the potassium ions (Bajpai et al., 2013; Tao et al., 2014). The concentration of free potassium ions in the suspensions of *P. digitatum*, *P. italicum*, and *G. candidum* mycelia was measured at 0, 3, 6, 9, 12, 24, and 48 h of treatment. The extracellular potassium concentration were determined in the supernatant using flame atomic absorption spectroscopy (Shimadzu AA6300, Japan).

The release of cellular constituents into the supernatant was measured according to a method described previously (Paul et al., 2011) with minor modifications. The release of cellular constituents into the supernatant was measured using a wavelength of 260 nm from a Multiskan Spectrum microplate spectrophotometer. Fungi was incubated in an environmental shaking incubator for 48 h, then the mycelia were collected and washed three times with phosphate buffered saline (pH 7.0) and resuspended in buffered saline. BP21 (10 or 100 μmol L^-1^) was added to the suspensions, and controls without BP21 were tested similarly. The results were expressed in terms of the optical density of absorption at 260 nm at 0, 3, 6, 9, 12, 24, and 48 h of treatment.

### Fruit Decay Tests

Experiments were carried out on freshly harvested navel oranges [*Citrus sinensis* (L.) Osbeck]. Fruit were harvested from a local orchard (Beibei, Chongqing). A previously described method was used in this experiment (Wang et al., 2018). Briefly, the fruit were surface-disinfected for 2 min in 2% sodium hypochlorite solution, washed and allowed to air dry. Citrus fruit were wounded (3 mm wide and 4 mm deep) by making punctures at two sites around the equator. The inocula contained 10^4^ CFU mL^-1^ spores and peptide BP21 at 8 μmol L^-1^ in water. As described in Section “Results,” two different times (A: 0 h; B: 16 h) of incubation of conidia with BP21 prior to inoculation were evaluated. Citrus fruit inoculated with conidia alone as the controls. The disease incidence (DI) and the lesion diameter (LD) was assessed daily. Three replicates (15 fruits per replicate, 2 wounds per fruit) were prepared for each treatment. The mean values ± SD of the DI and LD for each treatment were calculated.

### Hemolytic Activity of BP21

The hemolytic test was carried out by using 2% erythrocyte suspension, which was prepared from human blood according to the previous description (Muñoz et al., 2006) with partial modifications. No hemolysis and 100% hemolysis were determined for controls with normal saline (NS) and 0.1% Triton X-100, respectively. The AMPs BP21 (The final concentration was 8, 16, 32, or 64 μmol L^-1^) was mixed with 2% red blood cells and incubated at 37°C for 1 h. After diluted 600 times, commercial Prochloraz was mixed with erythrocyte suspension. The samples were centrifuged at 1,000 ×*g* for 5 min, and the supernatant was transferred to 96-well plates. Release of hemoglobin was determined by OD_540_, and the data were measured by a Multiskan Spectrum microplate spectrophotometer. The hemolytic activity of peptide was calculated as the percentage of total hemoglobin released compared with that released by incubation with 0.1% Triton X-100.

### Statistical Analysis

In the statistical analysis of the randomized complete block design, each treatment involved three replications, and the entire experiment was conducted in triplicate. The data were analyzed via a one-way analysis of variance (ANOVA), followed by Duncan’s multiple-range tests at *p* < 0.05 (SPSS Statistics 22.0, Inc.).

## Results

### Growth Inhibition of the Fungi by BP21 *in vitro*

**FIGURE 1:**
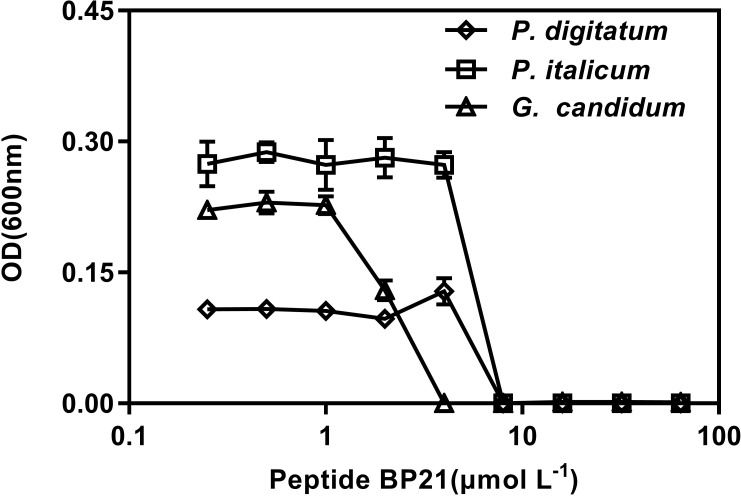
Dose–response curves of the growth inhibition of *Penicillium digitatum*, *Penicillium italicum*, and *Geotrichum candidum* by BP21 *in vitro*. The data shown are the mean values of the OD measurements at each peptide concentration after 48 h of incubation for *P. digitatum*, *P. italicum*, and *G. candidum*. Vertical bars indicate the standard error of the means.

The *in vitro* growth inhibition activities of the peptide BP21 were tested (**Figure 1**). The peptide BP21 showed the best inhibitory activity toward these three fungi. The MIC of BP21 against *P. digitatum*, *P. italicum*, and *G. candidum* was 8, 8, and 4 μmol L^-1^, respectively.

### Effect of BP21 on Morphological Alterations of Fungal Mycelia Analyzed Using Scanning Electron Microscopy (SEM)

**FIGURE 2:**
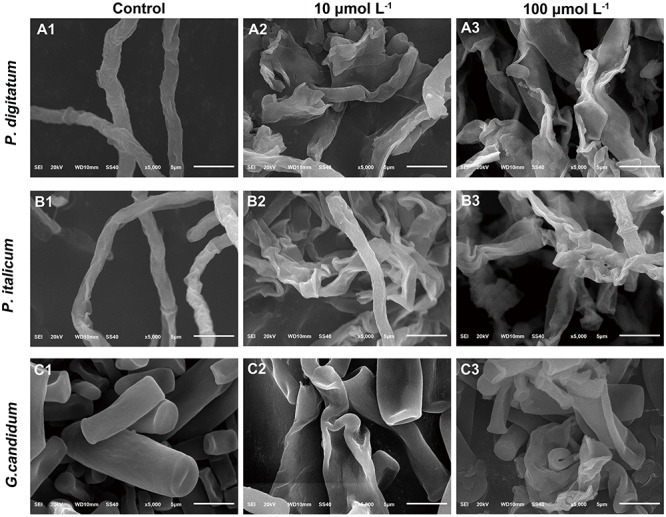
Scanning electron microphotography (SEM) of *P. digitatum* **(A)***, P. italicum* **(B)**, and *G. candidum* **(C)** mycelia treated with BP21. Mycelia were incubated in 5% PDB without BP21 **(A1–C1)** or with BP21 at final concentrations of 10 μmol L^-1^ **(A2–C2)** or 100 μmol L^-1^ **(A3–C3)**. Bars = 5 μm.

A SEM analysis was carried out to further visualize the effect of BP21 on the morphology of *P. digitatum, P. italicum*, and *G. candidum* mycelia, compared to control group (**Figure 2**). The control fungus without BP21 exhibited a regular and smooth surface (**Figures 2A1,B1,C1**). In contrast, *P. digitatum, P. italicum*, and *G. candidum* mycelia treated with BP21 (10 and 100 μmol L^-1^) exhibited considerable changes in hyphal morphology. The mycelia treated with BP21 appeared to be severely collapsed due to leak. Mycelia became deformed, shrunken, and distorted (**Figures 2A2,B2,C2,A3,B3,C3**). Increasing concentrations of BP21 resulted in more serious damage.

### Effect of BP21 on the Permeation of Fungal Mycelia Analyzed Using Fluorescence Microscopy

**FIGURE 3:**
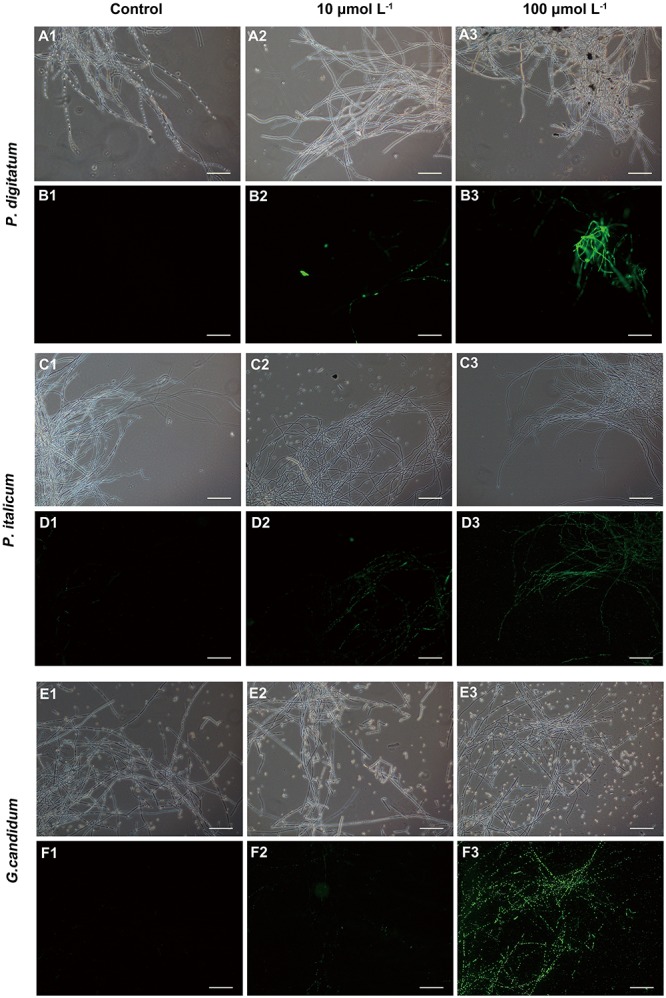
Fluorescence microscopy analysis of *P. digitatum* **(A,B)***, P. italicum* **(C,D)**, and *G. candidum* **(E,F)** mycelia treated with BP21. Mycelia were incubated in 5% PDB without BP21 **(A1–F1)** or with BP21 at final concentrations of 10 μmol L^-1^ **(A2–F2)** or 100 μmol L^-1^ **(A3–F3)**. Bars = 100 μm.

We used fluorescence microscopy and fluorescent dye SG to observe the mode of action of the mycelia with the peptide BP21. In controls in which mycelia was incubated with the SG probe without pretreatment with peptide BP21 (0 μmol L^-1^), no appreciable SG green fluorescent signal was discerned by using fluorescence microscopy (**Figures 3B1,D1,F1**). Mycelia exhibited slight discontinuous green fluorescence at 10 μmol L^-1^ BP21 (**Figures 3B2,D2,F2**). At this high BP21 concentration (100 μmol L^-1^), SG green fluorescence staining was very intense all along the *P. digitatum* and *P. italicum* mycelia (**Figures 3B3,D3**). *G. candidum* mycelia exposed to BP21 at 100 μmol L^-1^ exhibited discontinuous green fluorescent (**Figure 3F3**).

### The Effect of BP21 on Extracellular Conductivity of Fungal Mycelia

**FIGURE 4:**
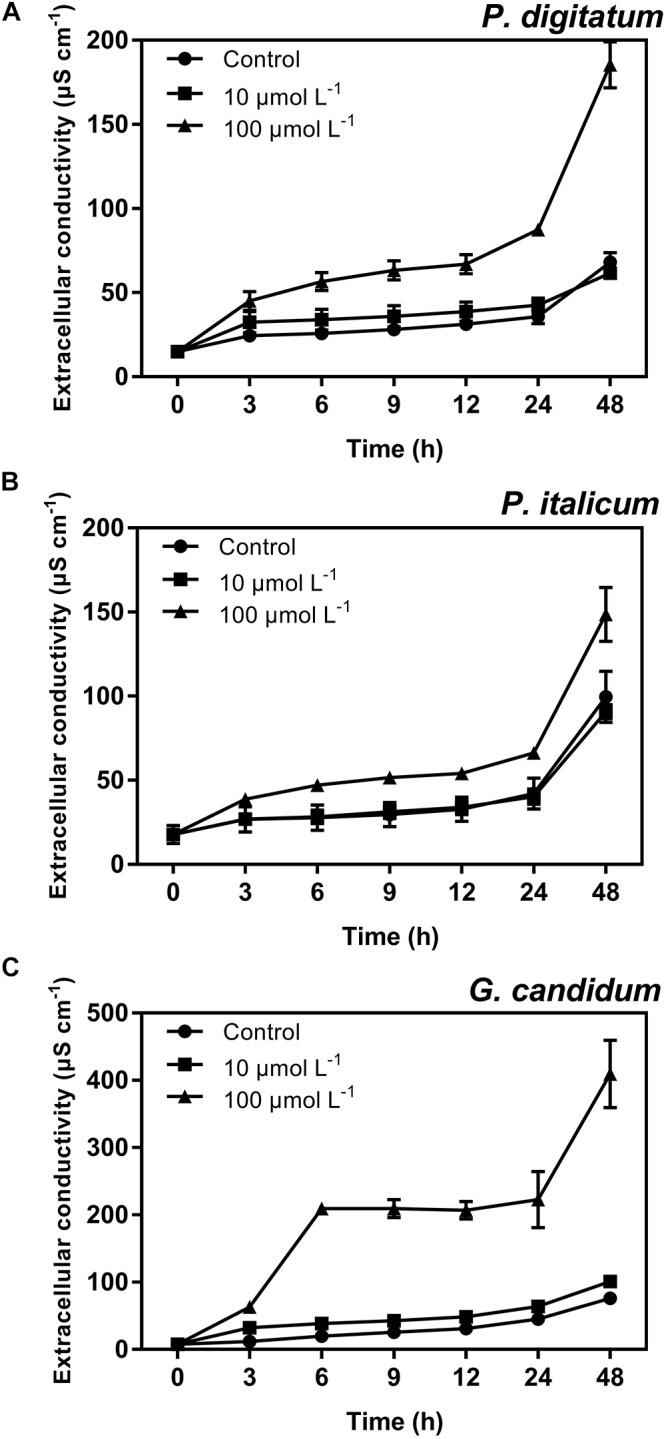
Extracellular conductivity of *P. digitatum* **(A)***, P. italicum* **(B)**, and *G. candidum* **(C)** mycelia treated with BP21. Mycelia were incubated in 10 or 100 μmol L^-1^ or without BP21 (control) solutions. Vertical bars indicate the standard error of the means.

Further antibacterial mode of action of peptide BP21 against the fungi was confirmed using the assay for the extracellular conductance (**Figure 4**). In this assay, the conductivity of all test groups increased gradually with increased treatment duration. The extracellular conductance sharply increased in the high-concentration (100 μmol L^-1^) BP21 treatment group (*p* < 0.05). According to the results, the peptide BP21 could increase the extracellular conductivity of *P. digitatum*, *P. italicum*, and *G. candidum*.

### Effect of BP21 on K^+^ Efflux of Fungal Mycelia

**FIGURE 5:**
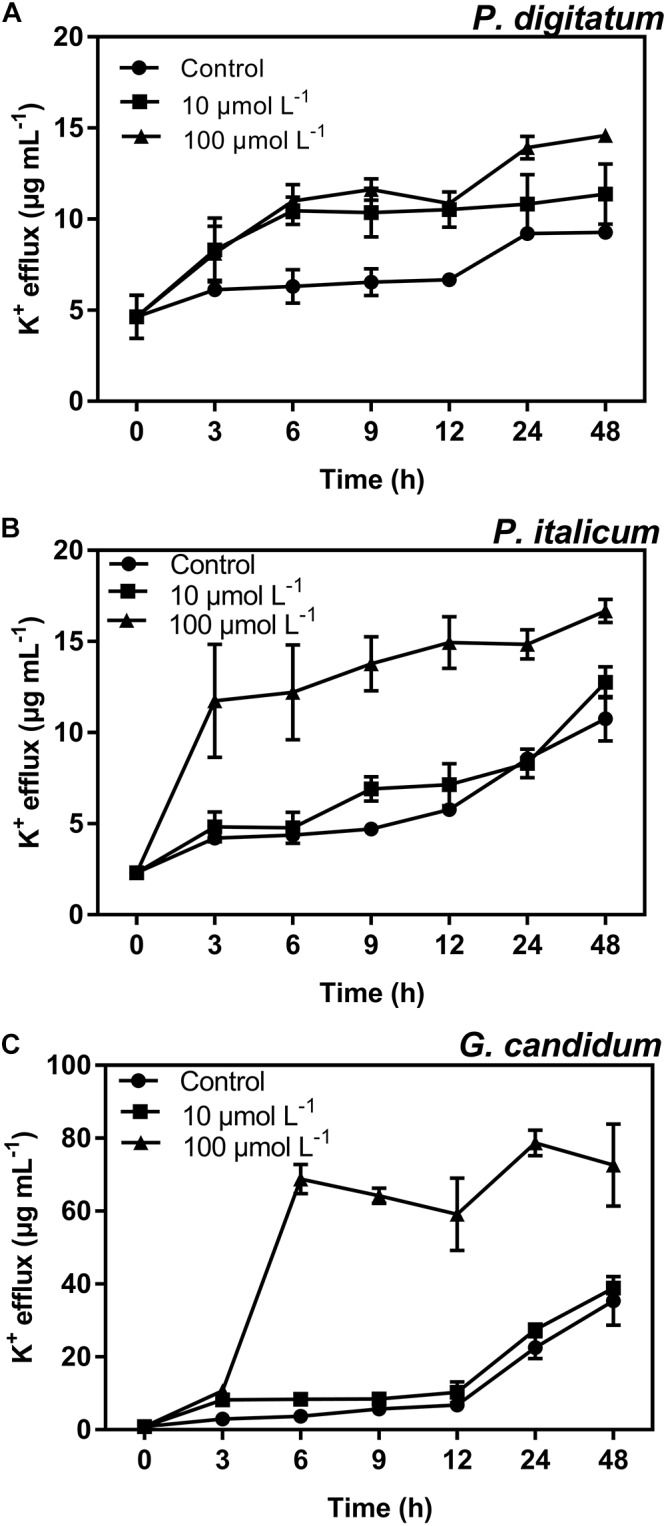
K^+^ efflux of *P. digitatum* **(A)***, P. italicum* **(B)**, and *G. candidum* **(C)** mycelia treated with BP21. Mycelia were incubated in 10 or 100 μmol L^-1^ or without BP21 (control) solutions. Vertical bars indicate the standard error of the means.

Potassium ions (K^+^) were found to leak from mycelia incubated with BP21 (**Figure 5**). BP21 significantly induced the release of K^+^, as the K^+^ efflux of the high-concentration (100 μmol L^-1^) BP21 treatment group was significantly higher (*p* < 0.05) than that of the control. Incubation with 10 μmol L^-1^ BP21 did not result in a K^+^ release significantly different from that of the control mycelia for *P. italicum* and *G. candidum* mycelia, but this concentration of BP21 did induce the release of K^+^ from *P. digitatum* mycelia. Moreover, *G. candidum* mycelia incubated with 100 μmol L^-1^ BP21 displayed significantly increased K^+^ release compared to the 10 μmol L^-1^ BP21 treatment group and the control group.

### Effect of BP21 on the Release of Cellular Constituents of Fungal Mycelia

**FIGURE 6:**
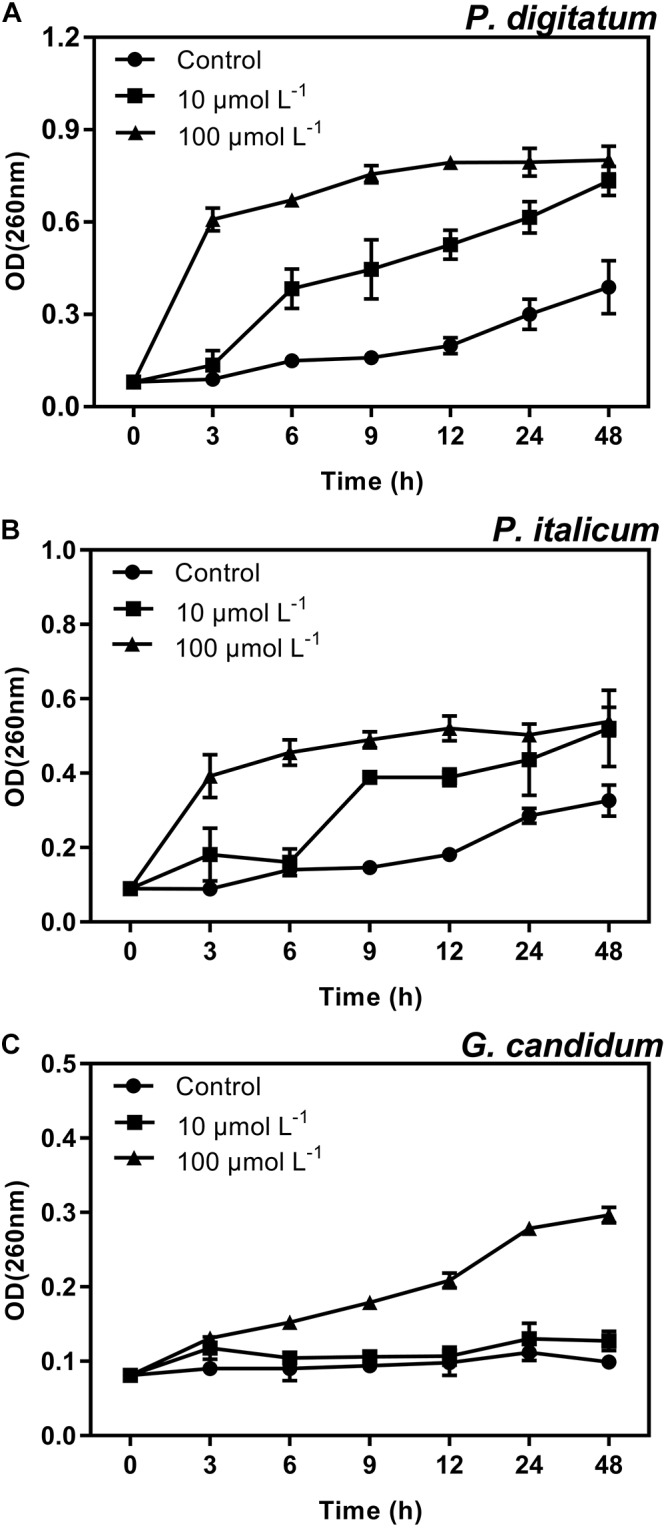
Release of cellular constituents of *P. digitatum* **(A)***, P. italicum* **(B)**, and *G. candidum* **(C)** mycelia treated with BP21. Mycelia were mixed with peptide BP21 at 10 or 100 μmol L^-1^ or without BP21 (control) in phosphate buffered saline. Vertical bars indicate the standard error of the means.

Another strategy for determining the mode of action of BP21 against these three filamentous phytopathogenic was to analyze the release of 260 nm absorbing materials from the treated mycelia of *P. digitatum, P. italicum*, and *G. candidum*. The OD_260_ value of the culture filtrates of *P. digitatum, P. italicum*, and *G. candidum* mycelia exposed to BP21 revealed an increasing release of cellular constituents with respect to exposure time (**Figure 6**). However, the OD_260_ values of untreated (control) mycelia of *P. digitatum* and *P. italicum* increased slowly, and only slight changes in the OD_260_ value of the untreated (control) mycelia of *G. candidum* were observed. This finding directly confirms the release of cellular constituents from *P. digitatum, P. italicum*, and *G. candidum* treated with BP21.

### Effect of BP21 on *P. digitatum, P. italicum*, and *G. candidum* Infections on Citrus Fruit

The inhibitory activity of the peptide BP21 against *P. digitatum*, *P. italicum*, and *G. candidum* infection was evaluated. The results showed that BP21 (treatment groups A and B) significantly inhibited citrus fruit diseases at 8 μmol L^-1^ compared to the non-treated controls (*p* < 0.05) (**Table 1**). Treatment B resulted in the most effective control of the three fungi growth on citrus fruit, wherein the growth of green mold and blue mold was reduced by 90% or more. Treatment A was more effective than the control treatment, but it was not as beneficial as treatment B. It turned out that the antifungal activity of BP21 increased along with time of incubation. In addition, BP21 completely controlled the *G. candidum* infection on citrus fruit, the LD and DI % were 0 (A and B). This could be indicated that BP21 could also control infection *in vivo*.

**Table 1:** Effects of BP21 on the fungal infection of citrus fruits.

Pathogen	Days	DI (%)			LD (mm)		
		Control	A	B	Control	A	B
*P. digitatum*	3	70.00 ± 10.00 a	6.67 ± 5.77 b	0.00 ± 0.00 b	18.57 ± 2.23 a	1.30 ± 1.17 b	0.00 ± 0.00 b
	4	100.00 ± 0.00 a	73.33 ± 11.55 b	3.33 ± 5.77 c	60.43 ± 3.61 a	25.75 ± 9.07 b	1.42 ± 2.45 c
	5	100.00 ± 0.00 a	86.67 ± 5.77 b	3.33 ± 5.77 c	95.15 ± 2.75 a	58.28 ± 8.62 b	2.67 ± 4.62 c
*P. italicum*	4	30.00 ± 17.32 a	16.67 ± 5.77 b	0.00 ± 0.00 c	5.32 ± 1.50 a	2.92 ± 0.88 b	0.00 ± 0.00 c
	5	79.17 ± 8.78 a	70.00 ± 10.00 a	0.00 ± 0.00 b	20.04 ± 0.83 a	13.82 ± 0.36 b	0.00 ± 0.00 c
	6	93.33 ± 11.55 a	86.67 ± 11.55 a	3.33 ± 5.77 b	33.24 ± 3.35 a	26.00 ± 0.98 b	0.25 ± 0.43 c
*G. candidum*	8	13.33 ± 15.28 a	0.00 ± 0.00 b	0.00 ± 0.00 b	3.43 ± 3.45 a	0.00 ± 0.00 b	0.00 ± 0.00 b
	10	16.67 ± 11.55 a	0.00 ± 0.00 b	0.00 ± 0.00 b	6.80 ± 2.22 a	0.00 ± 0.00 b	0.00 ± 0.00 b
	12	16.67 ± 11.55 a	0.00 ± 0.00 b	0.00 ± 0.00 b	9.81 ± 4.29 a	0.00 ± 0.00 b	0.00 ± 0.00 b


*Fruits were inoculated with conidia alone as control. Conidia were mixed with 8 μmol L^-*1*^ BP21 and either immediately inoculated (A) or incubated for 16 h prior to inoculation (B). Values are mean ± SD. The letters ‘a,’ ‘b,’ and ‘c’ indicate significant differences at the 0.05 level. The analysis was conducted using the data from the same pathogen on the same day.*

### Hemolytic Activity of BP21

**FIGURE 7:**
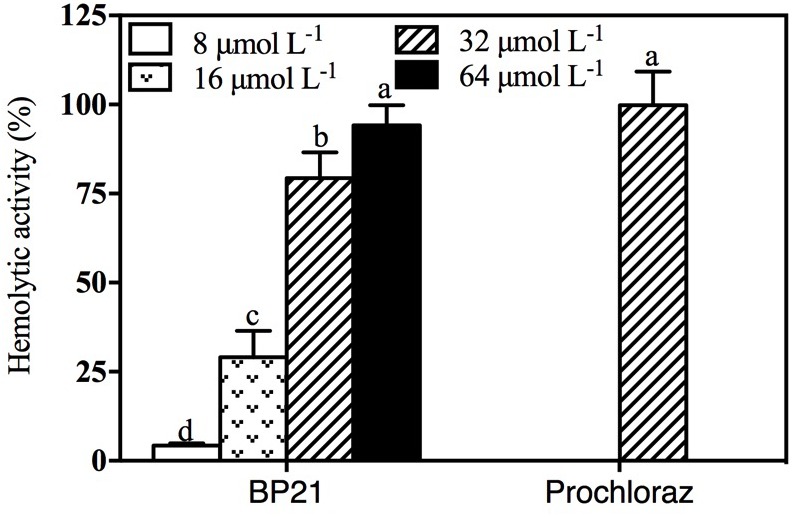
Hemolytic activity of BP21. Release of hemoglobin was determined by the value of OD_540_. The hemolytic activity is given as the mean ± SD of the percentage of human erythrocyte hemolysis. Values followed by different letters are significantly different according to Duncan’s multiple range test at *P* < 0.05.

Toxicity of different concentrations of BP21 to eukaryotic cells was determined by lysing human red blood cells (erythrocytes) (**Figure 7**). Prochloraz showed very high hemolytic activity, about 99.8%. Low concentration of BP21 showed low hemolysis, the hemolysis activity of BP21 at 8 μmol L^-1^ was 4.30%. However, the higher the concentration, the higher the hemolysis activity.

## Discussion

Blue mold and green mold are the primary post-harvest pathogen of citrus, and there is little effective control measures for sour rot; sour rot could be controlled by low-temperature environment, but chilling injury still causes major bottlenecks (Mercier and Smilanick, 2005). Therefore, exploration of effective methods for controlling these diseases have become urgently needed. Currently, the post-harvest application of short synthetic AMPs is an attractive alternative to fungicides (López-García et al., 2002).

In the present study, BP21 was shown to effectively inhibit the growth of *P. digitatum*, *P. italicum*, and *G. candidum in vitro.* When the concentration of BP21 was 8 μmol L^-1^, these fungi could not grow (**Figure 1**). The results of the SEM analysis clearly showed the difference between the treated and untreated fungi mycelia. The mycelia treated with BP21 became shrunken, collapsed, distorted, and formed a rough surface (**Figure 2**). This effect on the cell membranes of pathogens is similar to the effect of several essential oils (Helal et al., 2007; Bajpai et al., 2013) and citral (Tao et al., 2014). The effect may be attributed to the leakage of intracellular constituents. The cell membrane plays an important role in cell life activities, and cell membrane breakage causes the leakage of small molecular substances and ions. SG signals showed that BP21 could change the membrane permeability (**Figure 3**). The SG signals were more strong at higher concentration groups than at lower concentration groups. The increase in BP21 concentration resulted in a concomitant increase in the damage to the cell membrane. This mode is very similar to many of the cationic AMPs, such as some PAFs (Harries et al., 2013; López-García et al., 2015) and tetralipopeptides (Makovitzki et al., 2006), their mode of action involves permeation and disintegration of membranes. Membrane permeability parameters, including extracellular conductivity (**Figure 4**), leakage of potassium ions (**Figure 5**), and release of cellular constituents (**Figure 6**), were used to indicate gross and irreversible damage to the cytoplasmic and membranes (Bajpai et al., 2013). These parameters visibly increased as the concentration of BP21 increased. Although, hemolytic activity was positively correlated with the concentration of BP21 (**Figure 7**). After systematic toxicity evaluation in future research, BP21 could also be used at lower concentrations or be modified to reduce its hemolysis.

The peptide BP21 was shown to effectively control of *P. digitatum*, *P. italicum*, and *G. candidum* infection in citrus fruits *in vivo* (**Table 1**). Conidia were incubated with BP21 at a single concentration (8 μmol L^-1^) for 0 h (treatment A) or 16 h (treatment B) before inoculation. Treatment B resulted in the best performance of control of fungi infect and growth on fruits. Treatment A was more effective than the control treatment, but it was not as effective as treatment B. Obviously, in treatment B, the BP21 had more time to interact with the conidia, thus resulting in better disease control. This finding suggests that BP21 could be applied in production by soaking the fruits in a BP21 solution for a short duration. More research on the most effective method is warranted.

## Conclusion

The results of this study have shown that BP21 could effectively control infectious fungal diseases of citrus fruits, and it underlines the potential utility of BP21 as a novel broad-spectrum fungicide against pathogens of citrus as well. The major challenge of the widespread use of peptides for food and agriculture is to meet the requirement of a low production cost. Therefore, it is necessary to find or design peptides with no or little toxicity that control bacteria and fungi even when applied at low concentrations.

## Author Contributions

KZ conceived and supervised the project. WW and SL designed the experiments and performed most of the experiments. WW analyzed the data and wrote the manuscript. LD, SY, and JM gave advice and edited the manuscript. All authors read and approved the final manuscript.

## Conflict of Interest Statement

The authors declare that the research was conducted in the absence of any commercial or financial relationships that could be construed as a potential conflict of interest.
